# Spheronized drug microcarrier system from canola straw lignin

**DOI:** 10.1080/14686996.2022.2158369

**Published:** 2023-01-04

**Authors:** Liming Zhang, Antonia Svärd, Ulrica Edlund

**Affiliations:** a College of Textile and Clothing, Qingdao University, Qingdao, Shandong, China; b Fibre and Polymer Technology, KTH Royal Institute of Technology, Stockholm, Sweden; c AIMES - Center for the Advancement of Integrated Medical and Engineering Sciences, Department of Neuroscience, Karolinska Institute, Stockholm, Sweden

**Keywords:** Lignin, microparticles, canola straw, rapeseed, coumarin 153, ciprofloxacin

## Abstract

Inhomogeneous lignin from a canola (rapeseed) straw was isolated and valorized as regularly shaped spherical microparticles for drug delivery formulations. Lignin with a purity of 83% and broad molecular weight distribution (Ð > 5.0) was extracted by alkali pulping and acetylated to increase spheronization ability. Lignins with high degrees of acetylation (0.76 and 0.89) were successfully assembled into microparticles with uniform sizes (approximately 2 μm) and smooth spherical surfaces via solvent–antisolvent precipitation. Hydrophobic coumarin 153 and positively charged ciprofloxacin were used as model drugs to assess the encapsulation and release performance of lignin microparticles. Highly acetylated lignin microparticles displayed encapsulation efficiencies of 89.6% for coumarin 153% and 90.6% for ciprofloxacin. Scanning electron microscope images showed that coumarin 153 was encapsulated in the hydrophobic core, while ciprofloxacin was adsorbed on the less hydrophobic shell. The synthesis of lignin microcarriers not only provides a facile approach to utilizing waste canola straw lignin for drug delivery matrices but also has the potential to serve as an alternative lignin powder feedstock for bio-based materials.

## GRAPHICAL ABSTRACT

**Figure:**
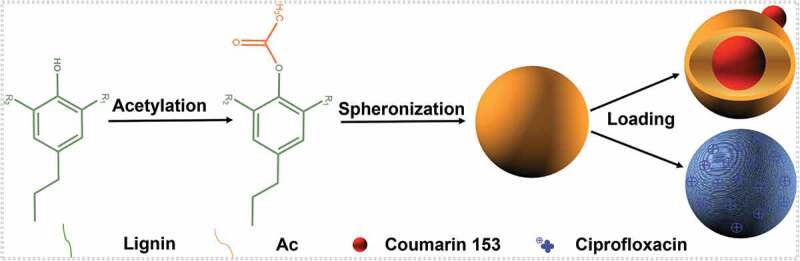

## 1. Introduction

Lignin is a vastly abundant yet widely underutilized renewable resource with high potential commercial value. However, its efficient utilization, either as a polymer or as a feedstock for lower molecular weight derivatives, is currently problematic because of the irregular morphology and inhomogeneity of lignin. Lignin is an irregularly branched polyphenolic polyether with three primary monolignol building blocks: p-coumaryl alcohol, coniferyl alcohol, and sinapyl alcohol. Structural units are connected via different kinds of ether linkages (C-O-C) and aromatic/non-aromatic C – C bonds in the lignin polymer, which involve mainly guaiacyl (G) and syringyl (S) units, and, in some plants, smaller amounts of p-hydroxyphenyl (H). Lignin structure and composition vary depending on the biomass origin and extraction method. Grass lignins are quite different from lignin found in perennial plants, such as wood lignin [1]. Moreover, lignin is typically isolated from lignocellulosic biomass in the pulping industry, mostly used for energy recovery and yet a largely untapped byproduct of material valorization. The isolation processes alter the native structure, morphology, and particle size of lignin due to irreversible condensations and the cleavage of chemical linkages [2,3]. Such treatments complicate the structure of native heterogeneous lignins and alter molecular weight distributions.

Different approaches have been used to obtain relatively uniform lignin with narrow molecular weight distribution and regular morphology, such as membrane filtration, solvent fractionation, and modification [3–7]. Duval et al. recently developed a solvent screening strategy to extract relatively uniform lignin from irregular samples of industrial softwood lignin [3]. Refined lignin exhibiting solubility in ethyl acetate had a narrow molecular weight distribution and was further used to synthesize thermosetting epoxies with tunable glass transition temperatures (T_g_) [3,8]. However, complex procedures and large amounts of organic solvents are typically required. Some researchers have focused on regular morphology, overcoming the high inhomogeneity of lignin by transforming lignin powder into lignin particles. The morphology of lignin can be greatly improved in this way, and nanostructured materials are expected to have considerably different properties than larger particles of the same composition [9]. Diverse approaches to producing submicron-scale lignin particles are presented in the literature. Dry and wet milling, spray drying [10], interfacial crosslinking [11,12], and self-assembly via solvent media (emulsion-solvent diffusion, emulsion solvent evaporation, and precipitation) [12,13] are conventional methods often used to fabricate lignin particles. A solvent-based process was recently developed to synthesize lignin particles from alkali lignin of wheat pulping black liquor [14] and Kraft lignin of softwood [15]. The process is simple to operate, and it involves the dissolution of lignin in a solvent and then the addition of an antisolvent to force lignin particles to form via self-assembly. Moreover, the particle size can be controlled by the type and concentration of lignin chosen [14,15].

Spherical lignin micro- or nanoparticles were demonstrated to have superior properties to general lignin powders, such as excellent surface activity, high UV shielding [16], strong resistance to oxidation, and antimicrobial activity [17]. Richter et al. made a composite of lignin nanoparticles with silver ions, and the resulting composite material exhibited higher antibiotic activity than silver nanoparticles [18]. Lu et al. used a solvent-based method to prepare nanoscale lignin particles with improved antioxidative and free radical scavenging activities and enhanced reducing power compared to bulk lignin [19]. Recent studies have shown that lignin nanoparticles are promising biodegradable carriers for drug delivery [20]. Other studies have verified the safety and feasibility of lignin incorporation into cells for potential pharmaceutical applications. For example, Tortora et al. demonstrated that lignin microcapsules are not cytotoxic and are readily incorporated in cells [21]. Furthermore, most drugs are water insoluble, requiring the carrier to improve their compatibility with water-soluble biological media [11]. Lignin is commonly considered a three-dimensional amorphous polymer comprising both hydrophobic and hydrophilic groups [22], which has advantages in drug transport and compatibility with biological media. Also, lignin has potential antioxidant and antimicrobial properties, and the structure can vary over a broad span depending on source and isolation process. For these reasons, lignin is rapidly gaining increasing interest from the scientific community as a potential controlled delivery carrier [23,24].

Canola, also known as rapeseed, is extensively cultivated in Europe as an oleaginous crop. Oil canola is an annual plant of high commercial importance, primarily as a source of seeds used for food oil, feed, and biodiesel. The remaining residues include rough and edgy straw, which, in comparison to wheat and rye straw, is not suitable as feed or bedding for livestock. In many cases, it is left on the field to retain some nutrients and capitalize on its soil-sanitizing effect for crop rotation. Straw residue can hence be considered a high-volume renewable resource with multiple benefits. A practical application of canola straw components would result in a double benefit, not only for economic profit but also in terms of biomass resource efficiency. The main components of canola straw are lignin, cellulose, hemicelluloses, and pectin, and the lignin percentage is more than 20% [25,26]. Canola straw lignin more resembles grass lignins and differs from cereal straw in having no detectable H-mers and an S:G ratio of ca. 1:0.7 [25,27]. Wheat and rye lignin typically consist of a small amount of H and ~1:1 in S:G unit ratio [28]. Previous studies have shown that it has a lower purity and a broader molecular weight distribution than wood lignin [25–27]. These irregular characteristics bring greater challenges in the utilization of canola lignin (CL) than wood lignin and applications are yet to be demonstrated.

We aimed to prepare spheronized lignin microparticles and explore their potential as biomaterial microcarriers and delivery agents for drugs, striving to overcome lignin inhomogeneity (mainly morphology) while making value-added products from canola straw lignin. Lignin was first extracted from canola straw by alkali pulping and then acetylated to increase spheronization capacity. Lignin microparticles were prepared via a solvent–antisolvent precipitation method. Two different drugs, coumarin 153 and ciprofloxacin, were incorporated, serving as hydrophobic and positively charged model drugs to assess the encapsulation and release performance of the lignin microparticles.

## 2. Experimental section

### 2.1 Materials

Canola straw (hybrid Compass) was collected from a farm in Österhaninge in Sweden. The collected straw was dried for 72 h at 40°C to a dryness of approximately 95% and stored cold and in the dark until use. Acetic anhydride (Ac_2_O, 99.0%) and ciprofloxacin (98%) were purchased from Fluka Chemie, Germany. Coumarin 153 (99%) and phosphate-buffered saline (PBS) tablets were purchased from Sigma-Aldrich. PBS buffer with pH = 7.4 was prepared by dissolving one PBS tablet in 200 mL of distilled water. Tetrahydrofuran (THF, 99.8%), and pyridine (99.5%) were purchased from Merck KGaA, Germany. Sodium hydroxide (NaOH, 98.5%), dimethylsulfoxide (DMSO, 99.9%), sodium dodecyl sulfate (SDS, proteomics Grade), chloroform-*d* (99.8%), and hydrochloric acid (HCl, 37%) were purchased from VWR International and used without further purification.

### 2.2 Soda pulping of canola straw and precipitation of lignin

CL was obtained from a canola straw through our in-house developed two-step soda pulping process (Figure S1, Supplemental material) [25]. Canola straw (100 g, dry weight) was placed in a 2.5 L steel autoclave, and air was evacuated from the system with a pump for 30 min. Then, 1 L NaOH (1.5 M) was added into the system by vacuum suction. The autoclaves were subsequently immersed in a steam-heated ethylene glycol bath equipped with a rotating device. The first extraction time was 60 min, and an extra 10 min was added to the beginning of extraction to allow the autoclaves to reach the set temperature of 110°C. At the end of the first extraction, 500 mL liquid was filtered out from the mixture, which was rich in hemicellulose. The solid and liquid residues were used to extract lignin at 155°C for 60 min. The extraction was terminated by cooling the autoclaves in a water bath. After 20 min, the black liquid from the second extraction was separated from the straw by filtration. The extracted liquid from the second extraction was freeze-dried for 48 h to isolate CL.

### 2.3 Acetylation of CL

**Figure 1.**
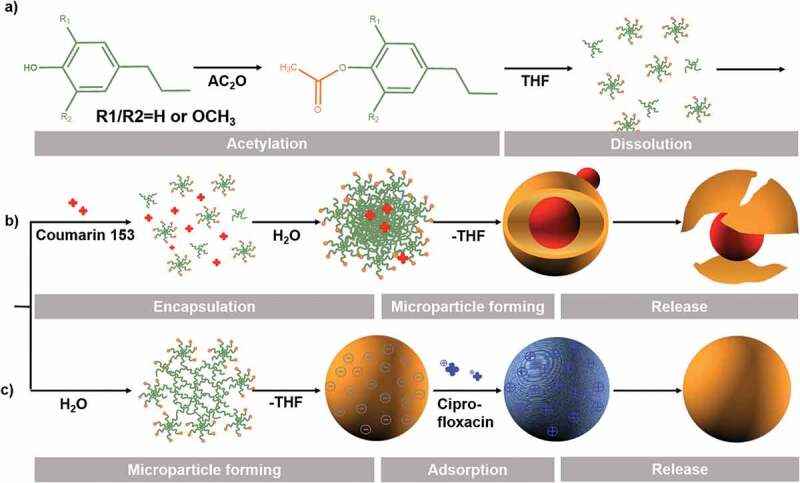
Schematic representation of lignin acetylation, the microparticle formation process, coumarin 153 and ciprofloxacin loading, and release of incorporated substances.

CL was acetylated according to a published procedure [29] with minor modifications. Specifically, 1 g lignin was weighed into a pre-dried reaction tube, to which 0.44 g of anhydrous pyridine and DMSO were added until the liquid:solid ratio was 3.5. The reaction mixture was stirred for 15–20 min, and 0.44 g Ac_2_O was added. The mixture was stirred at 20ºC for 8 h, after which the mixture evaporated to remove acetic acid and excess pyridine. Then, the residual liquid of the reaction mixture (the product in DMSO) was transferred into a fivefold excess of acidic water (pH = 2, adjusted by 1.0 M HCl) and washed during stirring. The washing step was repeated twice to produce refined acetylated lignin. The reaction was duplicated several times and is schematically outlined in Figure 1.

The yield in % was calculated as:

(1) $$Yield=ACL\left(g\right)/CL\left(g\right)\;*\;100$$

The degree of acetylation (DA) was calculated according to the following equation:

(2) $$DA=\left[CL\left(OH\right)-ACL\left(OH\right)\right]/\left[CL\left(OH\right)\text{\textbackslash{}break}+CL\left(OH\right)\;*\;ACL\left(OH\right)\;*\;0.042\right]$$

where CL(OH) (mmol/g) and ACL(OH) (mmol/g) represent the total concentrations of hydroxyl groups (Al-OH, Ph-OH, and COOH) in CL and acetylated canola lignin (ACL), respectively, as measured by ^31^P nuclear magnetic resonance (NMR). The coefficient 0.042 was calculated as the weight gain after acetylation (OH from the starting CL was converted to acetyl groups).

### 2.4 Preparation of lignin microparticles and microparticles loaded with coumarin 153 or ciprofloxacin

ACL microparticles loaded with coumarin 153 were prepared by dissolving ACL (20 mg/mL) and coumarin 153 (0.08 mg/mL) in 100 mL THF, and the mixture was placed in an ultrasonic bath (Branson Ultrasonic 5800, USA) for 10 min. Then, 80 mL acidic water (pH = 5, adjusted by HCl) was added in a dropwise fashion to the lignin/THF solution. Subsequently, the emulsion was centrifuged at 10,000 rpm for 10 min (Megafuge 8, Thermo Scientific, USA), and the precipitate was washed twice with distilled water. ACL microparticles loaded with ciprofloxacin were prepared in the same manner but with an acidic ciprofloxacin solution (0.5 mg/mL, pH = 5 adjusted by HCl) instead of acidic water. In parallel, ACL microparticles were prepared according to this protocol but without the addition of any drugs. Microparticles from unmodified CLs, with and without incorporated drugs, were prepared as reference samples.

The yield of lignin microparticles in % was calculated as:

(3) Yield(CL)=microparticles(g)/CL(g) *100

(4) Yield(ACL) =microparticles(g)/ACL(g)*100

The loading efficiency in % was calculated as:

(5) $$\begin{array}{l} \text{Efficiency=amount}\text{of}\text{loaded}\text{drug}(\text{g)}\\ \;\;\;\;\;\;\text{/amountofaddeddrug}(\text{g})\text{*}\text{100} \end{array}$$

### 2.5 Drug release

Lignin microparticles (30 mg) loaded with coumarin 153 were added to 15 mL of SDS solution (5% w/v). After 30 min of incubation, the suspension was centrifuged at 10,000 rpm for 10 min. The centrifuged microparticles were suspended in 15 mL of fresh SDS solution, and the supernatant was filtered to measure coumarin 153 release. The concentration of released coumarin 153 was determined by UV−vis detector (Shimadzu UV-2550, Japan) at 412 nm. The procedure was repeated several times until no UV absorption attributable to coumarin 153 was observed. The generated calibration curve had a correlation coefficient (*R*^2^) of 0.994.

Ciprofloxacin release experiments were performed on stoppered vials containing 30 mg of microparticles and 15 mL of PBS solution. The test vials were maintained at 37°C in an orbital shaker incubator (Gallenkamp) with 50 oscillations per min for 3 days. Aliquots (0.8 mL) of release medium were collected from the reaction mixture at different intervals for analyses of drug concentrations. The ciprofloxacin concentration in each aliquot was measured via UV−vis absorption maximum at 276 nm using a generated calibration curve with a correlation coefficient (*R*^2^) of 0.998.

All release experiments were performed in triplicate.

### 2.6 Characterization

**Figure 2.**
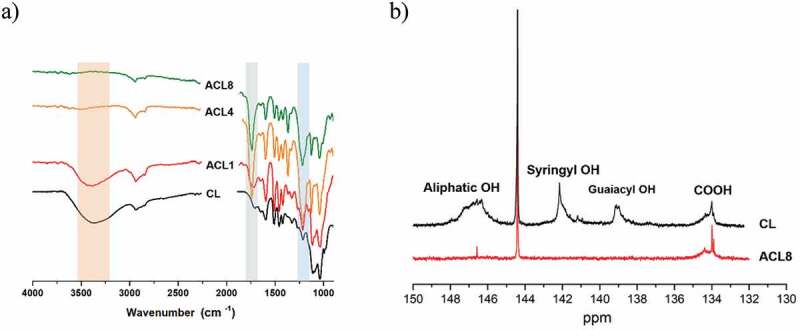
(a) FTIR transmittance spectra and (b) ^31^P-NMR of CL and ACLs.

The number of hydroxy groups in lignin was evaluated using ^31^P-NMR with a procedure based on the method of Argyropoulos [30]. Briefly, lignin was reacted with 1,3,2 dioxaphospholanyl chloride so that hydroxyl groups are phosphorylated and can be detected and quantified by ^31^P-NMR. Freshly prepared samples were dissolved in chloroform-*d* (25 mg/mL) and immediately analyzed with ^31^P-NMR at room temperature using a Bruker 400 MHz NMR spectrometer. The following NMR parameters were used: 256 scans, 4 dummy scans, and a relaxation time of 5 s. The assignments of ^31^P-NMR spectra were as follows: The signals of the aliphatic hydroxyls (Al-OH) and carboxyl groups (COOH) in lignin appeared in the regions 146.0–149.0 and 134.2–135.5 ppm, respectively. The peaks of the guaiacyl phenolic OHs (Ph-OH) and the syringyl Ph-OH were separately located at 138.8–140.2 and 142.2–143 ppm, respectively. NMR data are shown in Table 2 and Figure 2.

**Table 1.** Sample abbreviation, average molecular weight, yield, and DA of CL and ACL.

Sample	AC: OH^a^	M_w_ (g/mol)	M_n_ (g/mol)	Ð ^b^	Yield (%)^c^	DA
CL	-	6720	1242	5.4	-	-
ACL0.5	0.5:1	6881	1360	5.1	49.2 ± 2.3	0.24
ACL1	1:1	7324	1363	5.4	50.6 ± 1.7	0.44
ACL2	2:1	7503	1506	5.0	42.8 ± 1.5	0.50
ACL4	4:1	7882	1529	5.2	67.5 ± 1.8	0.76
ACL8	8:1	7194	1360	5.3	72.0 ± 2.0	0.89

^a^AC:OH = acetic anhydride:OH of canola lignin, n/n.

^b^Ð = M_w_/M_n_.

^c^compared with the weight of CL.

**Table 2.** Quantification of the functional groups (mmol/g) in CL and ACL samples by quantitative ^31^P-NMR.

Sample	Aliphatic OH	Phenolic OH	COOH	Total (mmol/g)
CL	2.13	1.82	0.44	4.39
ACL0.5	1.44	1.43	0.31	3.18
ACL1	1.04	0.95	0.30	2.29
ACL2	1.06	0.64	0.29	1.99
ACL4	0.32	0.36	0.25	0.93
ACL8	0.06	0	0.37	0.43

The molecular weight of the lignin was analyzed by an SEC 1260 infinity (Polymer standard service, Germany) equipped with a PSS precolumn, a PSS 100 Å column, and a PSS GRAM 10,000 Å analytical column coupled in series. The samples were prepared by dissolving 4 mg of lignin in 2 mL of DMSO containing 0.5% (w/w) LiBr. The solutions were stored at 60°C in a oven overnight and passed through 0.45 μm syringe filters before injection. Molecular weights were calculated based on the retention time of pullulan standards (molecular weights 342−708,000 g/mol, PSS, Germany).

FTIR spectra were recorded using a Perkin Elmer Spectrum 2000 spectrometer (Perkin Elmer Instruments, Inc., UK) equipped with a single reflection attenuated total reflectance (ATR) accessory (golden gate) from Graseby Specac (Kent, UK). Each spectrum was recorded as an average of 16 scans at a resolution of 4 cm^−1^ in the range 4000–1000 cm^−1^, and corrections were made for atmospheric water and CO_2_.

Microparticle morphology was characterized by FE-SEM (Hitachi SEM S-4800, Japan). Silica slices were cleaned with ethanol and a plasma cleaning system before use. Samples were prepared by dropping colloidal dispersions onto silica slices and then drying at 25°C for 24 h. The dried slices were mounted on carbon tape-coated stubs and sputter-coated with a 2.7 nm thick layer of gold/palladium under an inert atmosphere using a Cressington 20 HR Au/Pd sputter coater.

The Z-averages of lignin microparticles were measured with a Nano Zetasizer (Malvern Instrument, UK) with a 633 nm He-Ne laser at 25°C. Each sample was analyzed three times, and the average values are reported.

## 3. Results and discussion

### 3.1 Lignin recovery from canola straw

CL was obtained through our in-house developed two-step soda pulping process in which the first extraction was designed to extract hemicelluloses, and the second extraction was applied to obtain lignin [25]. The lignin extraction process is schematically represented in Figure S1 (Supplemental material). Lignin produced with this method was sulfur-free, unlike commercial lignin from sulfite pulping, and to a lesser extent, Kraft pulping. The composition of CL was thoroughly investigated in our recent study and consisted of lignin 83%, glucose 1%, and xylose 15%, (relative percentages only considering lignin and monosaccharides, w/w) [25]. The ash content was 9% in total. The purity of CL was lower than that of similar systems reported in the literature [25,26]. Table 1 reports the recorded molecular weights and dispersities of CL, and Figure S2 (Supplemental material) shows the molecular weight distribution curves. CL fractions had a relatively large weight-average molecular weight (M_w_ 6720 g/mol) and a broad dispersity (Ð=M_w_/M_n_, >5). Lignin isolated from canola was reported to have a low weight-average molecular weight (M_w_ 3500–4500 g/mol) and a narrow dispersity (<2.0) when a hydrothermal or dilute acid pretreatment was added to the extraction process [26].

### 3.2 Synthesis of ACL

Figure 1(a) illustrates the synthesis of ACL. Acetylated lignin does exist naturally in lignocellulosic biomasses such as that from kenaf bast, a few nonwoody plants, and hardwoods [31,32]. Natural acetates of lignin are, however, hydrolyzed and removed during alkaline isolation and degradative processes [33]. In the experiment, we adopted an alkaline extraction method to obtain the lignin sample, so the samples were mainly non-acetylated lignin. Non-acetylated lignin separated from wheat pulping black liquor did not form spheres with the ‘solvent and antisolvent self-assembly’ method, as reported by Qian et al. [14]. CL was, therefore, modified to increase the spheronization ability. Still, we purposely did not strive for full acetylation; partial acetylation is preferred to retain some chain hydrophilicity in favor of spheronization.

ACL samples were prepared with five different molar ratios to obtain different DAs and assess the impact of DA on the spheronization ability. A molar ratio of -OH in CL and Ac_2_O of 0.5:1 (ACL0.5) resulted in a DA of 0.24 (Table 1). The value of DA increased sharply, from 0.44 to 0.89, when the molar ratio was increased from 1:1 to 8:1. The molar ratio had a significant effect on the reaction. The acetylation reaction was consistently conducted at room temperature, and the effect of temperature was hence not investigated. Acetylation of lignin at higher temperatures (70–120°C) and without a catalyst was reported in the literature [34]. The reaction conducted at higher temperatures required a relatively short time but consumed considerable energy. Acetylation resulted in moderate increases in the molecular weights of CL samples (Table 1). All acetylated samples had a Ð of approximately 5. ACL 8 had the highest yield, 72.0 ± 2.0%.

### 3.3 Structural analysis of ACL

FTIR spectra and ^31^P NMR analysis clearly showed the structural changes in lignin after acetylation (Figure 2 and Table 2). FTIR of CL reveals a dominant band at approximately 3368 cm^−1^, originating from the O-H stretching vibration of aromatic and aliphatic moieties. However, the intensity of this band significantly decreased in all ACL samples, indicating the successful acetylation of the hydroxyl groups. This was especially true for ACL4 and ACL8, for which almost all the hydroxyl groups were substituted by acetyl groups. The acetylation reaction also gave rise to a strong absorption peak at 1705 cm^−1^, which is attributed to the conjugated/unconjugated carboxyl ester groups or unconjugated β-ketone carbonyl groups. Moreover, the band intensity at 1215 cm^−1^, which stems from C-C, C-O, and C=O stretching, increased in the acetylated samples. The bands at 1123 and 1032 cm^−1^ stem from the aromatic in-plane C-H bending deformation for syringyl- and guaiacyl-type lignin units, respectively, and were found in both CL and all ACL samples. The ^31^P NMR data (Figure 2 and Table 2) were consistent with the results from the FTIR analysis. CL exhibited sharp peaks for aliphatic OH, syringyl OH, guaiacyl OH, and COOH. ACL showed no signals for syringyl OH and guaiacyl OH, indicating substantial substitution of the OH groups during acetylation, and relatively weak signals for aliphatic OH and COOH.

Acetylation was also indicated by the color changes in the samples. As shown in Figure S3 (Supplemental material), CL had a dark black color, acetylated lignin ACL0.5 was dark brown, and ACL8 had a light-yellow color. The dark color of lignin originates from some specific chromophore and auxochrome groups. The main chromophore groups include quinone groups, conjugated double bonds, and carbonyl groups, while the auxochrome groups include phenolic hydroxyl groups, hydroxyl groups, and carboxyl groups. The acetylation reaction blocks the hydroxyl groups in lignin, resulting in a lighter color [35].

### 3.4 Lignin microparticles formation

Lignin microparticles were prepared by solvent–antisolvent precipitation. ACL4 gave the highest yield of microparticles, ~77%, whereas CL only yielded ca. 29% (Table 3). THF served as a solvent and acidic water as the antisolvent in the preparation of ACL microparticles. Due to the poor solubility of CL in THF (50% was insoluble), some samples were lost during precipitation and centrifugation, and the resulting yields were low. Acetylation of CL clearly increased the solubility in THF (Figure S3f, Supplemental material) and thus the yields of microparticles (Table 3). Z-averages (Table 3) indicate that higher DA seems to result in larger microparticles. Interestingly, the Z-average first decreased and then increased as the lignin:acetic anhydride molar ratios increased, which will be further discussed in the next section. The size is one indicator with which to determine if the lignin sample is suitable for the production of lignin microparticles, but the surface morphology of lignin microparticles is more important.

**Table 3.** Yield, Z-average, and dispersity of lignin microparticles.

Sample	Yield (%)	Z-average (nm)	Dispersity
CL	29 ± 2	2480 ± 280	0.50
ACL0.5	32 ± 3	3650 ± 260	0.57
ACL1	51 ± 2	2030 ± 300	0.66
ACL2	58 ± 3	2430 ± 190	0.53
ACL4	77 ± 3	3050 ± 160	0.46
ACL8	63 ± 4	3120 ± 120	0.44

### 3.5 Morphological analysis of lignin microparticles

**Figure 3.**
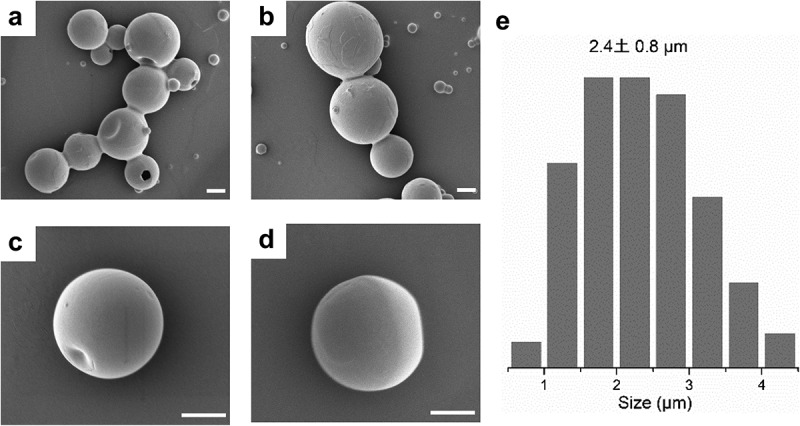
Scanning electron micrographs of the lignin microparticles obtained from ACL4 (a, c) and ACL8 (b, d). The white scale bar in each micrograph represents 1 μm. The inset diagram (e) shows the size distribution of ACL8 microparticles as calculated with SEM.

CL and ACL with a low DA value (0.24) did not form spheres (Figures S4 a and b, Supplemental material), while ACL4 (Figures 3(a,c)) and ACL8 (Figure 3(b,d)) particles had uniform spherical shapes with dense and smooth surfaces. The different spheronization abilities between CL and ACL may be explained by the gradual self-assembly of the lignin chains in the THF-H_2_O dispersion media [14]. When lignin was completely dissolved in THF, lignin aggregates disassociated into small lignin molecules, and these lignin molecules stretched in the solution. When antisolvent (H_2_O) was added to the homogeneous system, lignin aggregates were re-formed; hydrophobic chains formed cores, and less hydrophobic fractions formed shells. The lignin microparticles were thus formed via self-assembly in the solvent. ACL and CL are structurally different in the sense that most of the hydrophilic phenolic groups in CL were converted to hydrophobic ester groups in ACL. The fact that CL failed to produce colloid spheres may, therefore, be related to phenolic groups. First, the phenolic hydroxyl groups in CL might cause electrostatic repulsion, which prevents dense packing in sphere-shaped nanoparticles. Second, hydrophilic phenolic hydroxyl groups in CL may form hydrogen bonds between the lignin chain and water, which hinders colloid formation. This is in line with a similar study in which only fully acetylated lignins in THF formed colloidal spheres upon the addition of water. Qian et al. [14]. concluded that the self-assembly mechanism for lignin spheres is related to hydrophobic interactions, including van der Waals and π–π interactions. Compared with CL, ACL has tighter π–π interactions, stronger van der Waals interactions, weaker electrostatic repulsion, and less hydrogen bonding, contributing to the formation of microspheres.

The particle size distribution was calculated from SEM micrographs by image analysis. The average diameter of ACL8 microparticles was 2.4 ± 0.8 μm (Figure 3(e)). This value differs slightly from the size value (ACL8 Z-average, 3120 ± 120 nm) measured with DLS. This might be because the principles of the two methods are different. DLS measures fluctuations in the intensity of light scattered by Brownian motion, while SEM analysis gives the geometric diameter as a basic quantity. Furthermore, the samples are in dispersion in DLS and in the dry state in SEM. The differences in sizes measured by the two methods are more significant for samples with low DA. ACL0.5 exhibited a Z-average of 3653 nm. SEM clearly showed that ACL0.5 particles were not spherical, but were larger aggregates of irregular particles (Figure S5b, Supplemental material). Aggregation explains why ACL0.5, with a low DA, had such a high Z-average (nm) value. Therefore, for samples with a low DA, the Z-average value has no reference value, but for samples with a high DA (≥0.44), the Z-average value can reflect the size of the lignin microparticles very well. Table 3 indicates that higher DA seems to result in larger microparticles, with Z-averages of acetylated lignin samples ACL1, ACL2, ACL4, and ACL8 of 2030 ± 300, 2430 ± 190, 3050 ± 160, and 3120 ± 120 nm, respectively. This may be because the samples with higher DA have longer hydrophobic chains, which formed larger hydrophobic cores during the self-assembly process.

### 3.6 Drug loading of lignin microparticles

Coumarin 153 and ciprofloxacin were employed as model drugs to test the encapsulation and release properties of lignin microparticles. Coumarin 153 was chosen because of its relative hydrophobicity and fluorescence. It is widely used as a fluorescent marker *in vitro* and *in vivo* to visualize the cellular uptake of nanoparticles. Ciprofloxacin is a broad-spectrum antibiotic soluble in acidic water. Additionally, ciprofloxacin is a zwitterionic compound and exists in its cationic form at pH < 6.1, while it is anionic at pH > 8.7 [36]. With these two model drugs, we evaluated the potential of canola-derived lignin to serve as a drug carrier. The coumarin 153 encapsulation efficiency of CL was 15 ± 1%, while ACL microparticles had noticeably higher encapsulation efficiencies (Table 4); ACL8 had the highest value: 90 ± 1%. ACL microparticles were also more efficient than CL in the encapsulation of ciprofloxacin, again approaching 90% encapsulation for the ACL grade with the highest DA. Drug encapsulation caused the microparticles to increase in size. CL and ACL microparticles loaded with drugs (coumarin 153/ciprofloxacin) (Table 4) had larger Z-average values than corresponding lignin microparticles in the absence of drug loading (Table 3).

**Table 4.** Drug loading data.

Sample	Coumarin 153 encapsulation efficiency (%)	Z-average of microparticles with coumarin 153 (nm)	Ciprofloxacin adsorption efficiency (%)	Z-average of microparticles with ciprofloxacin (nm)
CL	15 ± 1	2860 ± 180	51 ± 2	3060 ± 200
ACL0.5	47 ± 1	3760 ± 200	40 ± 2	4050 ± 220
ACL1	55 ± 1	3950 ± 110	52 ± 2	3790 ± 180
ACL2	68 ± 2	4630 ± 140	62 ± 2	4760 ± 140
ACL4	79 ± 1	4930 ± 130	96 ± 1	4560 ± 160
ACL8	90 ± 1	5550 ± 150	91 ± 1	5170 ± 110

### 3.7 Drug release from lignin microparticles

**Figure 4.**
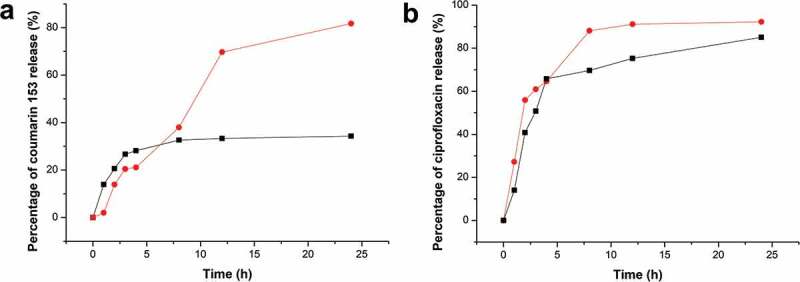
The release profiles expressed as % in released weight/weight of actually loaded drug (losses during preparation were not considered) for (a) coumarin 153 and (b) ciprofloxacin from microparticles of CL (■) and ACL8 (·).

The profiles for the release of coumarin 153 and ciprofloxacin from CL and ACL8 microparticles are shown in Figure 4. Both ACL8 and CL microparticles are released over 60% of the loaded coumarin 153 within 3.5 h at 20°C in water with 5% SDS. Initially, the amount of coumarin 153 released from ACL8 was lower than that released from CL microparticles. This was expected, considering that ACL8 formed regular microspheres; this may lead to the embedding of all coumarin 153 in the interior matrix, thereby prolonging release. In contrast, CL did not form microspheres (Figure S4a, Supplemental material) but minute irregular fragments, making the loaded coumarin 153 more exposed and easier to release promptly. After 2.5 h, ACL8 exhibited a much higher release efficiency (69.7 ± 1.5%) than CL. The accumulated release might be caused by the disintegration of spherical structures following erosion and mass loss resulting from the release of encapsulated drugs.

For ciprofloxacin, both CL and ACL8 microparticles exhibited an initial burst of release behavior, with a release of 88.1 ± 1.7% and 69.6 ± 2.8% within 8 h from ACL8 and CL, respectively. At 24 h, 92.2 ± 1.9% and 85.1 ± 2.7% of the ciprofloxacin had been released from ACL8 and CL, respectively. After 3 days, the cumulative release levels reached 93.7 ± 1.8% and 85.8 ± 2.1%.

### 3.8 Morphological analysis of drug-encapsulated microparticles and particles after release

**Figure 5.**
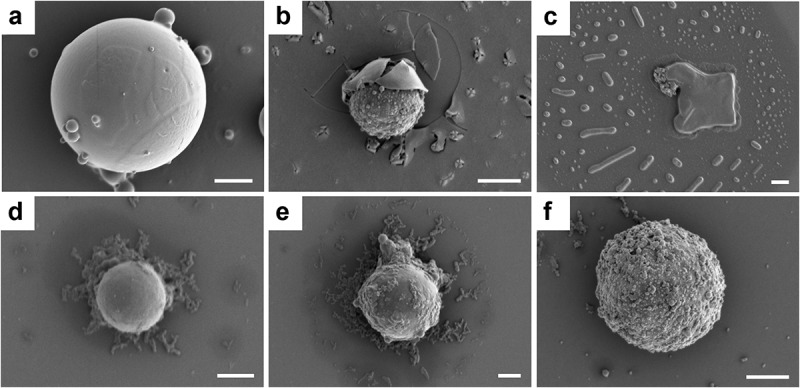
Scanning electron micrographs of ACL8: (a) loaded with coumarin 153 before release, (b) loaded with coumarin 153 after 3 h of release, (c) loaded with coumarin 153 after 10 h of release, d) loaded with ciprofloxacin before release, (e) loaded with ciprofloxacin after 5 h of release, and (e) loaded with ciprofloxacin after 10 h of release. The white scale bar in each micrograph represents 1 μm.

The morphologies of lignin microparticles encapsulated with drugs were observed with SEM before and after release (Figure 5). ACL8 microspheres encapsulating coumarin 153 were initially spherical and had smooth surfaces (Figure 5(a)). Several small spheres were attached to the large particles, which may be residual coumarin 153 not incorporated into the lignin microparticles. After incubation, and as the release progressed, a core–shell structure of the microparticles was revealed (Figures 5(b,c)). The structural organization and its role in drug release can be explained as follows: during the encapsulation process (Figure 1), coumarin 153 and lignin were completely dissolved in the THF solution after ultrasonication. Then, water was added, causing hydrophobic ACL molecules to start forming clusters. Coumarin 153 is hydrophobic and, to an increasing extent, it was transferred from solution to aggregates with ACL molecules as the water content was further increased. Hence, as sphere formation proceeded, coumarin 153 was embedded in the microparticles in which the previously established ACL clusters and coumarin 153 formed the cores of the spheres. There were likely no covalent linkages between the cores and the shells in the ACL spheres, so the cores and shells were separated during the subsequent drying process. SEM images of the microparticles taken after drug release (Figures 5(b,c)) clearly show the separation of the shell and core. The release of coumarin 153 was accelerated in a 5% (w/v) SDS solution because no significant release of coumarin 153 was observed from ACL microparticles in water suspension (Figure S5, Supplemental material). The addition of 5% SDS to the aqueous medium was expected to increase the affinity of coumarin 153 for the receiving medium and weaken the interactions between the drug molecules and the ACL matrix [11]. The SDS molecules interfered with the stabilizing properties of lignin at the core−shell boundaries and triggered the release of coumarin 153 because of the higher solubility of the hydrophobic molecule in the SDS solution. The drug release process was hence accompanied by the disintegration of the shell structure.

The morphologies of lignin microparticles loaded with ciprofloxacin looked quite different from the corresponding microparticles with encapsulated coumarin 153. Ciprofloxacin was absorbed on the surfaces of the lignin microparticles (Figure 5(d)). This might be because ciprofloxacin is soluble in acidic water and therefore associated with fewer hydrophobic-chain segments and remained in solution and at the solid–liquid interphase when lignin microparticles formed. When spherical particles formed, ciprofloxacin was adsorbed on the surface by electrostatic interactions. Moreover, lignin is negatively charged; ciprofloxacin, on the other hand, exists in its cationic form at pH < 6.1 (the pH in this experiment was 5). The negative charges in lignin may have stemmed from carboxylic groups, which could explain why ACL8 adsorbed a higher percentage of ciprofloxacin than CL [37]. The release profile for ciprofloxacin microparticles was also quite different from those of lignin microparticles encapsulated with coumarin 153, as shown in Figures 5(e,f). Ciprofloxacin was gradually released by erosion from the surface, which became rougher as the release proceeded (Figures 5(e,f)).

The cumulative release curves from Figure 4 were fitted by zero-order, first-order, Higuchi [38], Weibull [39], and Ritger-Peppas [40] equations, and the results are listed in Table S1, Supplemental material. The correlation coefficient (*R*^2^) values showed that the release of coumarin 153 from CL and ACL8 microparticles was better explained by Ritger-Peppas kinetics than by other models. The Weibull distribution model showed the best fit for ciprofloxacin release. The value of the release exponent (*n*) in the Ritger-Peppas equation varies according to different release mechanisms [38]. The obtained values suggest that coumarin 153 release from ACL microparticles was controlled by the erosion of the microparticles, while release from CL particles was caused by both Fickian diffusion and erosion. The *R*^2^ values of the ciprofloxacin release fit lines were generally lower than the *R*^2^ values for coumarin 153 release. The Weibull distribution showed a closer fit than the other models. This model only describes the curve in terms of the shape parameter but does not describe the kinetic properties of the drug [41].

From these results, we can conclude that the lignin microparticles consist of a hydrophobic core and a shell. The modes of encapsulation of coumarin 153 and ciprofloxacin into lignin microparticles are different. Coumarin 153 (representing a hydrophobic drug) was embedded inside the core, while ciprofloxacin (representing hydrophilic drugs with a positive charge) was adsorbed on the surface by electrostatic forces. The loaded drugs were released in different ways according to the mode of encapsulation, and the processes included rupture and release. The successful encapsulation of these two different drugs shows that lignin microparticles have broad potential to serve as delivery vehicles for poorly soluble drugs and as dispersants for positively charged drugs. Microcarriers in the size range produced are interesting for intramuscular, subcutaneous, or subdermal implanting, following consistency-regulating formulation. The shells of lignin microparticles can effectively protect confined drugs from the surrounding environment. The shell layer prevented and controlled the diffusion of the confined compounds from the core, providing a barrier with micrometer thickness. As shown in Figure S5 (Supplemental material), the lignin microparticles were re-dispersed in water very well. This work is a proof-of-concept study; cytotoxicity and immunological responses will be important to study before *in vivo* applications are considered. Canola lignin microparticles offer a structurally defined alternative to regular lignin powders, enable valorization of nonedible agricultural biomass, and provide a low-cost route to high-volume applications of robust lignin.

## 4. Conclusions

Inhomogeneous canola straw lignin was recovered through sulfur-free alkali pulping processing and used as a raw material to prepare relatively homogeneous lignin microparticles. Acetylation of CL significantly enhanced the capacity for spheronization. ACL (DA = 0.89) formed regular smooth microspheres (average diameter 2.4 μm) with hydrophobic cores and negatively charged hydrophilic shells, while non-acetylated lignin formed only irregular sub-micron fragments. The lignin microparticles were loaded with coumarin 153 and ciprofloxacin, which served as model drugs to reveal the potential for hydrophobic and positively charged drug delivery. Coumarin 153 was encapsulated primarily in the hydrophobic core of lignin and acetylated lignin particles and was released over 4 days with kinetics best described by the Ritger-Peppas model. Ciprofloxacin loading was characterized by adsorption onto the less hydrophobic shell of the lignin microparticles. Lignin microparticles showed potential as drug carriers for hydrophobic and positively charged drugs. However, its application scope, as well as toxicity and biocompatibility, will be discussed in further study. Microparticles from canola lignin offer a new approach to the production of value-added products from residual agricultural biomass and have the potential to be a structurally defined alternative to lignin powder.

## Supplementary Material

Supplemental Material

## Abbreviations

CL: canola straw lignin
ACL: acetylated canola lignin
T_g_: glass transition temperature
Ac_2_O: acetic anhydride
PBS: phosphate-buffered saline
THF: tetrahydrofuran
NaOH: sodium hydroxide
DMSO: dimethylsulfoxide
SDS: sodium dodecyl sulfate
HCl,: hydrochloric acid
SEM: scanning electron microscope
DLS: Dynamic light scattering
FTIR: Fourier transform infrared spectroscopy
NMR: Nuclear magnetic resonance spectroscopy
