# Supplementary material for: Spheronized drug microcarrier system from canola straw lignin
Source: Sci Technol Adv Mater. 2023 Jan 4;24(1):2158369. doi: 10.1080/14686996.2022.2158369 (PMC9828816; doi:10.1080/14686996.2022.2158369)
Supplement: Supplemental Material [file TSTA_A_2158369_SM0444.docx]

**Supplemental material**

**Spheronized drug microcarrier system from canola straw lignin**

Liming Zhang^a,b^, Antonia Svärd^b^, Ulrica Edlund^b,c*^

^a^ College of Textile and Clothing, Qingdao University, Ningxia Road 308, Qingdao, Shandong, 266101, China

^b^ Fiber and Polymer Technology, KTH Royal Institute of Technology, Teknikringen 56, Stockholm, 100 44, Sweden

^c^ AIMES - Center for the Advancement of Integrated Medical and Engineering Sciences, Department of Neuroscience, Karolinska Institute, 171 77 Stockholm, Sweden

Corresponding author: Ulrica Edlund, E-mail: edlund@kth.se, Tel:+46 8 7907634


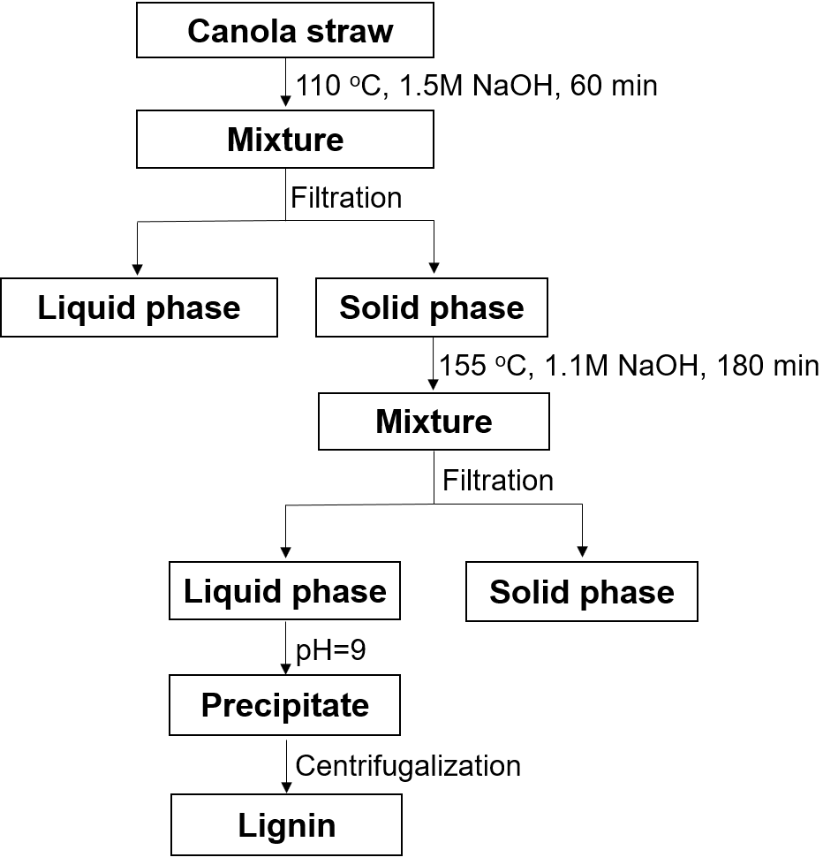


Figure S1. Extraction protocol for the recovery of lignin from canola straw.


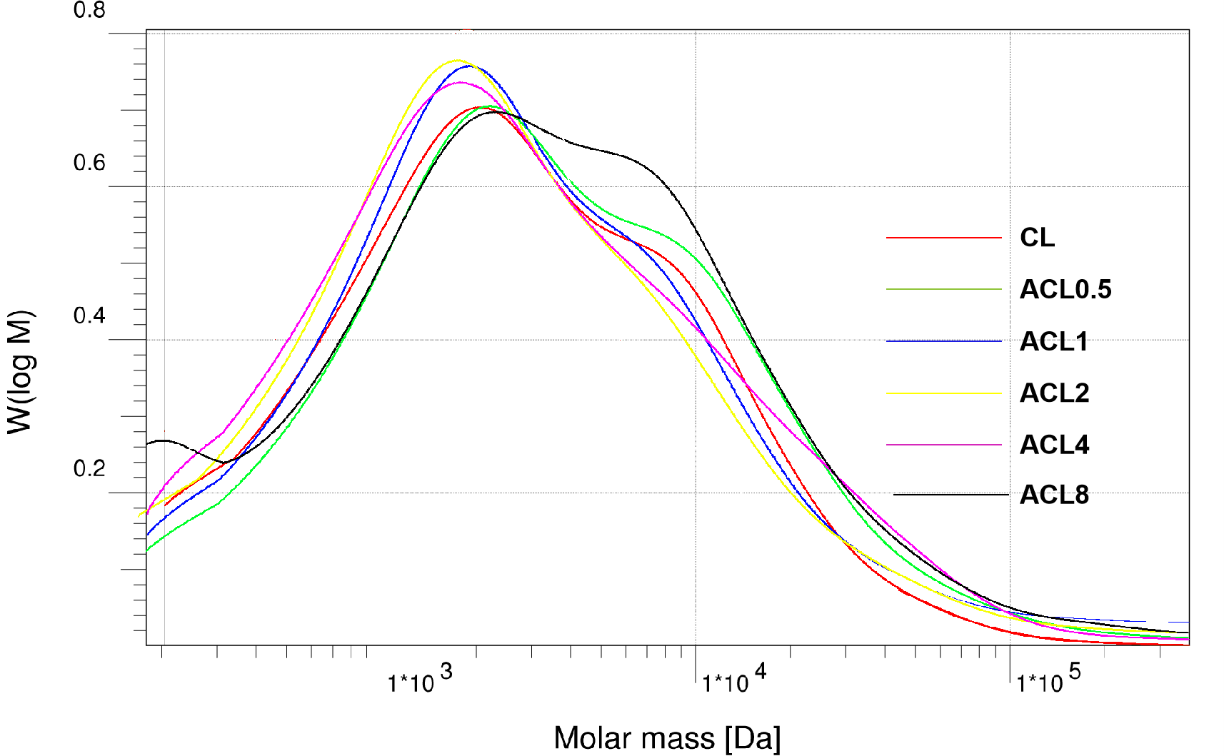


Figure S2. Molecular weight distribution of canola lignin (CL) and acetylated canola lignin ACL1, ACL2, ACL4, and ACL8.


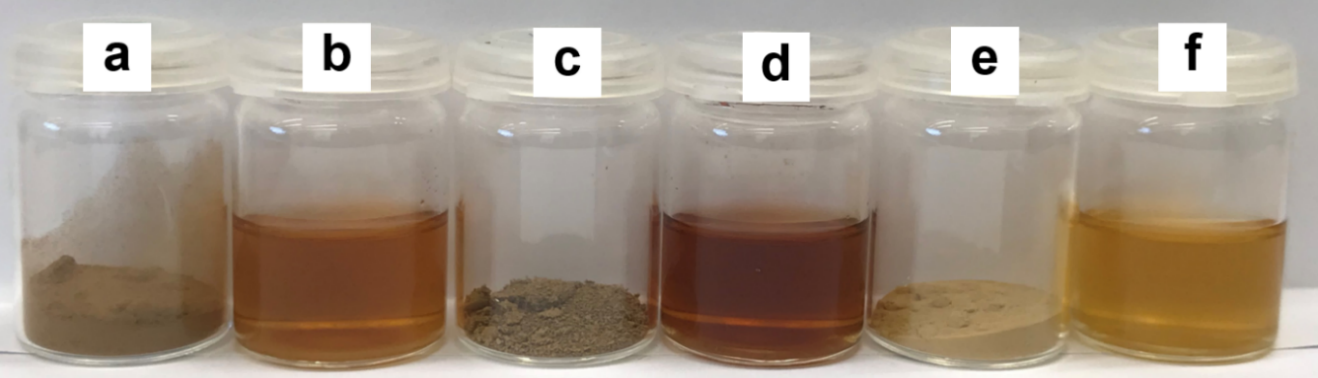


Figure S3. Photographs of a) CL, c) ACL0.5, e) ACL8, and their solutions, respectively, in THF (b, d, and f).


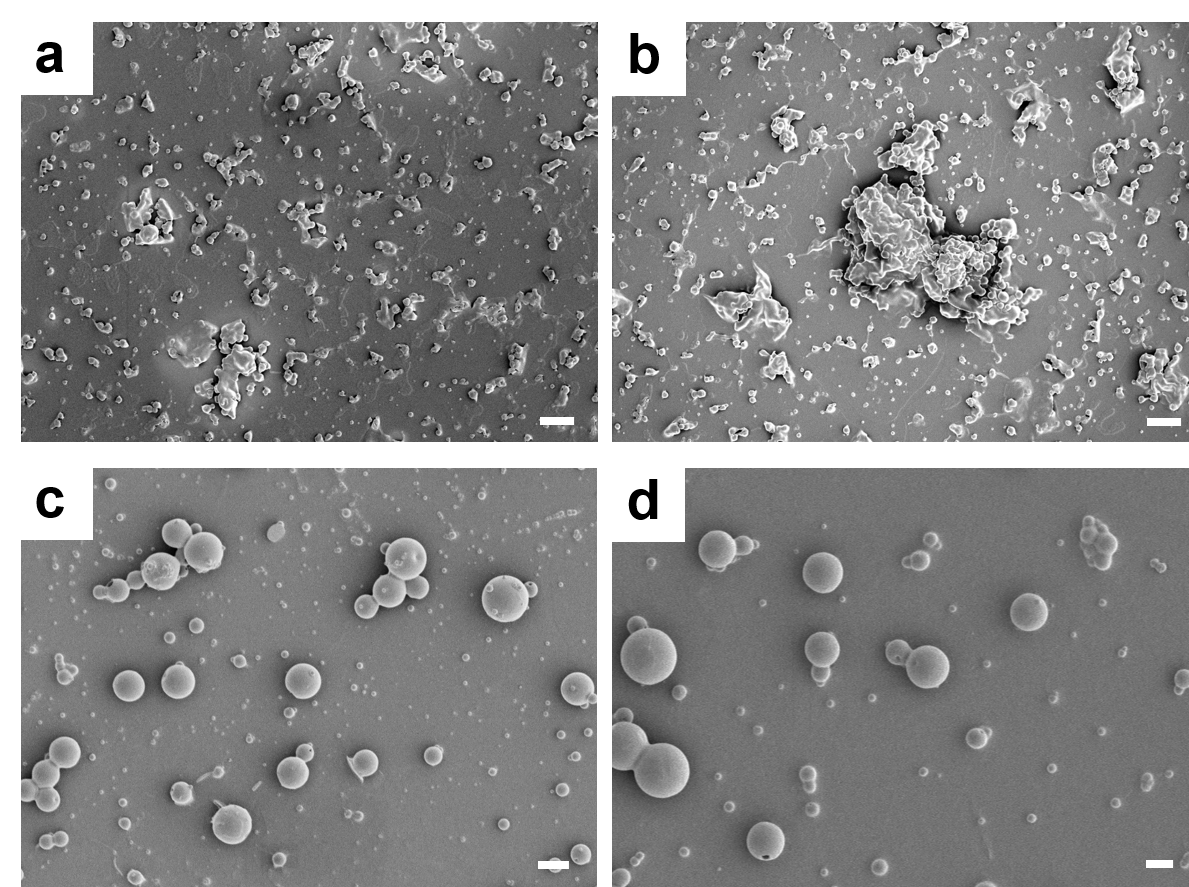


Figure S4. Scanning electron micrographs of a) CL, b) ACL0.5, c) ACL1, and d) ACL2 particles. The white scale bar in each micrograph represents 1 μm.


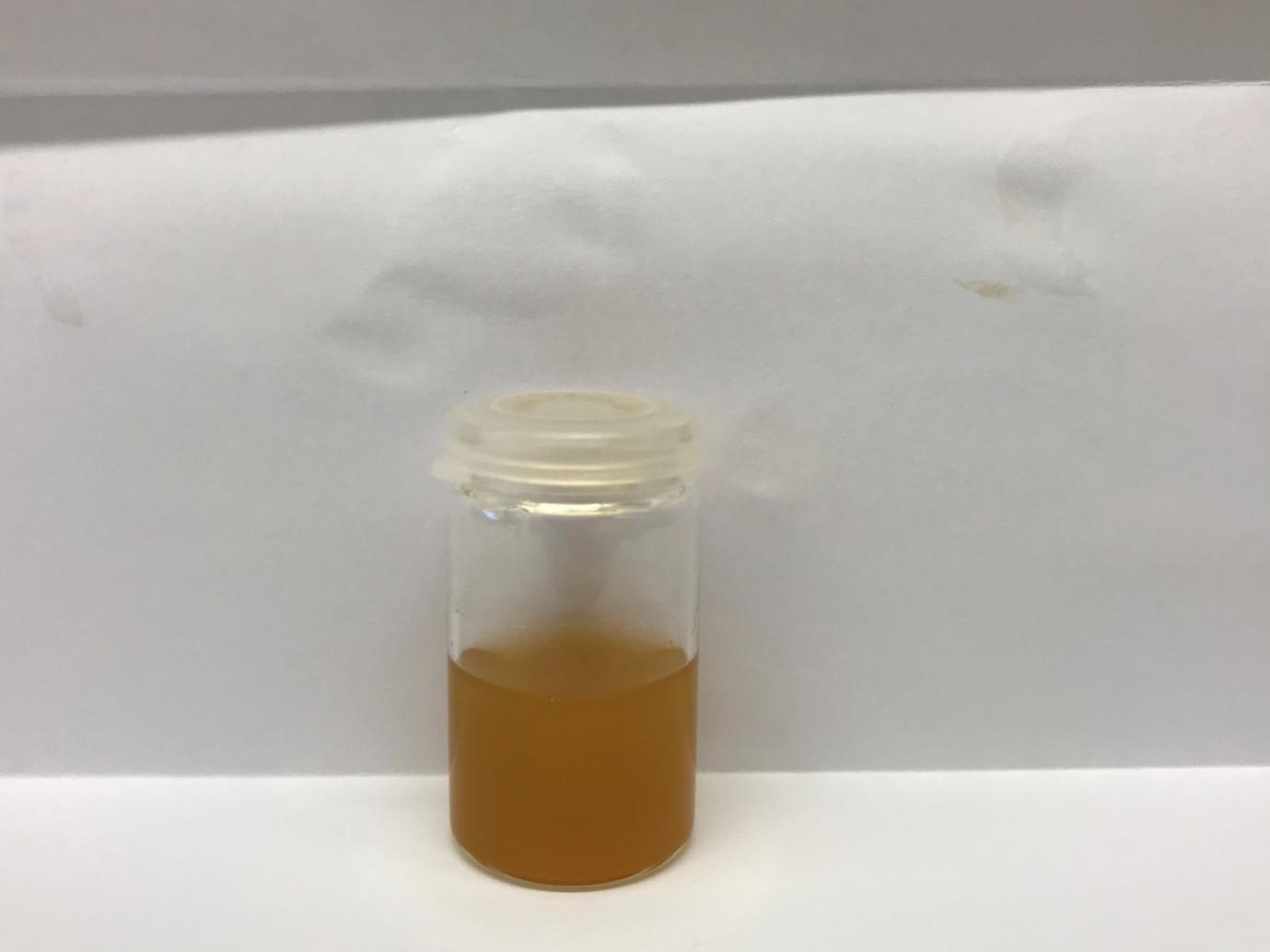


Figure S5. Lignin microparticles re-dispersed in water (ACL8 loaded with coumarin 153).

Table S1. Fit lines of drug release curves from canola lignin microparticles.

| Sample | Model | Kinetic equations | R^2^ |
| --- | --- | --- | --- |
| CL+ Coumarin 153 | Zero-order [1] | R_t_ = 0.1638t+0.0408 | 0.9658 |
|  | First-order [1] | ln(1-R_t_) =  -0.2934t+0.0764 | 0.9474 |
|  | Higuchi [1] | R_t_ = 0.446$\sqrt{t}$-0.2334 | 0.9423 |
|  | Weibull [2] | Ln(ln$\frac{1}{1-Rt}$)=0.472lnt+0.2852 | 0.8086 |
|  | Ritger-Peppas [3] | ln$\frac{Rt}{R\infty}$= 0.8023 lnt -15309 | 0.9677 |
| ACL8+ Coumarin 153 | Zero-order | R_t_=0.2533t-0.1588 | 0.9297 |
|  | First-order | ln(1-R_t_) =-0.5415t+0.5033 | 0.8748 |
|  | Higuchi | R_t_=0.6794$\sqrt{t}$-0.5681 | 0.8806 |
|  | Weibull | Ln(ln$\frac{1}{1-Rt}$) =2.0876lnt -2.3463 | 0.9547 |
|  | Ritger-Peppas | ln$\frac{Rt}{R\infty}$= 1.7273lnt -2.4372 | 0.955 |
| CL+ ciprofloxacin | Zero-order | R_t_=0.0063t+0.5102 | 0.375 |
|  | First-order | ln(1-R_t_) =-0.0199t-0.7988 | 0.5589 |
|  | Higuchi | R_t_=0.0753$\sqrt{t}$+0.366 | 0.5744 |
|  | Weibull | Ln(ln$\frac{1}{1-Rt}$)=0.5312lnt -1.1322 | 0.7724 |
|  | Ritger-Peppas | ln$\frac{Rt}{R\infty}$= 0.2091lnt -1.5553 | 0.7271 |
| ACL8+ ciprofloxacin | Zero-order | R_t_=0.0063t+0.5102 | 0.375 |
|  | First-order | ln(1-R_t_) =-0.0276t-1.188 | 0.4926 |
|  | Higuchi | R_t_=0.0704$\sqrt{t}$+0.49 | 0.537 |
|  | Weibull | Ln(ln$\frac{1}{1-Rt}$)=0.4927lnt -0.6576 | 0.8267 |
|  | Ritger-Peppas | ln$\frac{Rt}{R\infty}$ = 0.2536 lnt -0.879 | 0.7092 |

t= time

R_t_=cumulated drug release percentage

The cumulative release curves from Figure 4 were fitted by zero-order, first-order, Higuchi, Weibull, and Ritger-Peppas equations, respectively, and the results are listed in Table S2. The correlation coefficient (R^2^) values show that the release of Coumarin 153 was better explained by Ritger-Peppas kinetics in comparison to other models. In the Ritger-Peppas equation, the value of the release exponent (n) varies according to different release mechanisms. As shown in the table, CL has an n value of 0.8023, and ACL8 has a value of 1.7273. When n≤0.45, the drug release occurs by Fickian diffusion, n values between 0.45~0.89 indicate anomalous transport, while n≥0.89 indicates that the release is controlled by erosion. The obtained values suggest that the Coumarin 153 release from ACL microparticles was controlled by the erosion of the microparticles while release from CL particles was caused by both Fickian diffusion and erosion. It indicated that the acetylated lignin formed very spherical particles that the release occurred when the microspheres were eroded by 5% SDS. R^2^ values of the ciprofloxacin release fit lines were generally lower than R^2^ values for coumarin 153 release. Weibull distribution showed a closer fit than the other models. This model only describes the curve in terms of the shape parameter but does not describe the kinetic properties of the drug [4].

# REFERENCES

1. Dash S, Murthy PN, Nath L, et al. Kinetic modeling on drug release from controlled drug delivery systems. *Acta Poloniae Pharmaceutica* 2010; 67(3): 217-223.

2. Kosmidis K, Macheras P. On the dilemma of fractal or fractional kinetics in drug release studies: A comparison between Weibull and Mittag-Leffler functions. *International Journal of Pharmaceutics* 2018; 543(1-2) : 269-273.

3. Ritger PL, Peppas N A. A simple equation for description of solute release I. Fickian and non-fickian release from non-swellable devices in the form of slabs, spheres, cylinders or discs. *Journal of Controlled Release* 1987; 5(1) : 23-36.

4. Koester LS, Ortega GG, Mayorga P, et al. Mathematical evaluation of in vitro release profiles of hydroxypropylmethylcellulose matrix tablets containing carbamazepine associated to β-cyclodextrin. *European Journal of Pharmaceutics and Biopharmaceutics*, 2004; 58(1): 177-179.
